# Combining Spirometry and the ARISCAT Respiratory Risk Assessment Can Improve Postoperative Outcomes and Reduce Mortality Risk in Mexico

**DOI:** 10.1016/j.opresp.2024.100325

**Published:** 2024-04-18

**Authors:** Yolanda Mares-Gutiérrez, Adrián Martínez-González, Guillermo Salinas-Escudero, Manuel García-Minjares, Stephanie Liu, Yvonne N. Flores

**Affiliations:** a Pulmonary Physiology Department, Hospital General de México Dr. Eduardo Liceaga, C.P. 06720 Mexico City, Mexico; b Departamento de Investigación, Subdirección de Regulación y de Atención Hospitalaria, Dirección Médica, ISSSTE, C.P. 14050 Mexico City, Mexico; c Departamento de Salud Pública, Facultad de Medicina, Universidad Nacional Autónoma de México, C.P. 04510 Mexico City, Mexico; d Centro de Estudios Económicos y Sociales en Salud, Hospital Infantil de México Federico Gómez, C.P. 06720 Mexico City, Mexico; e Coordinación de Universidad Abierta, Innovación Educativa y Educación a Distancia, CUAIEED, Universidad Nacional Autónoma de México, C.P. 04510 Mexico City, Mexico; f Rosemead School of Psychology, Biola University, La Mirada, CA 90639, United States; g UCLA Department of Health Policy and Management, Fielding School of Public Health, Los Angeles, CA 90095, United States; h UCLA Center for Cancer Prevention and Control Research and UCLA-Kaiser Permanente Center for Health Equity, Fielding School of Public Health and Jonsson Comprehensive Cancer Center, Los Angeles, CA 90095, United States; i Unidad de Investigación Epidemiológica y en Servicios de Salud, Morelos, Instituto Mexicano del Seguro Social, Cuernavaca, Morelos C.P. 62000, Mexico

**Keywords:** Spirometry, ARISCAT, Postoperative complications, Mortality, Espirometría, Escala de riesgo respiratorio en pacientes quirúrgicos de Cataluña, Complicaciones posoperatorias, Mortalidad

## Abstract

**Introduction:**

Although a major goal of preoperative evaluation is to identify risk factors and improve postoperative outcomes, current clinical guidelines in Mexico indicate that preoperative spirometry should only be performed on patients with pulmonary disease. The aim of this study was to compare the incidence of postoperative complications (POC), mortality, and risk factors among adults who did or did not undergo preoperative spirometry, based on their Assess Respiratory Risk in Surgical Patients in Catalonia (ARISCAT) risk level.

**Material and methods:**

An observational, retrospective and comparative study design was used to identify 2059 patients from the *General Hospital of Mexico* who had an ARISCAT assessment during 2013–2017. Patients were classified in two groups: ARISCAT with spirometry (*n* = 1306) and ARISCAT without spirometry (*n* = 753). Chi-square, Fisher's exact test and the Student's *t*-tests were used to compare groups. Logistic regression was used to identify factors associated with an increased risk of POC and mortality.

**Results:**

In the ARISCAT with spirometry group, 11% of patients had POC, compared with 48% of patients in the ARISCAT without spirometry group. High-risk ARISCAT patients who did not receive spirometry had higher mortality (18%), than those who underwent spirometry (0.4%). Logistic regression results indicate that not performing preoperative spirometry increases the probability of POC and mortality.

**Conclusions:**

Our findings suggest that the combined use of preoperative spirometry and ARISCAT is associated with reduced POC and mortality. Future clinical guidelines should recommend the use of preoperative spirometry for patients with a moderate or high ARISCAT level in Mexico.

## Graphical abstract

**Figure:**
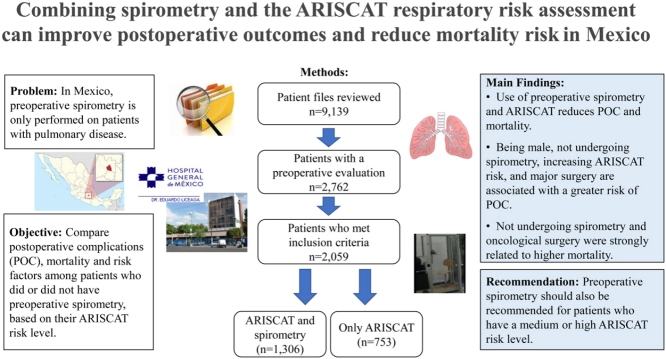

## Abstract (es)

**Introducción:**

La evaluación preoperatoria se centra en identificar los factores de riesgo y mejorar los resultados posoperatorios; las guías clínicas actuales en México indican la espirometría preoperatoria solo en pacientes con enfermedad pulmonar. El objetivo fue comparar la incidencia de complicaciones posoperatorias (CPO), la mortalidad y los factores de riesgo en pacientes clasificados por su nivel de riesgo de la escala de riesgo respiratorio en pacientes quirúrgicos de Cataluña (ARISCAT) sometidos o no a una espirometría preoperatoria.

**Material y métodos:**

Estudio observacional, retrospectivo y comparativo. Se clasificaron 2.059 pacientes del Hospital General de México durante 2013 a 2017; en dos grupos: ARISCAT con espirometría (n = 1.306) y ARISCAT sin espirometría (n = 753). Se aplicaron pruebas de *X*^2^, *f* de Fisher y *t* de Student para comparar los grupos y análisis de regresión logística para identificar los factores asociados a mayor riesgo de CPO y mortalidad.

**Resultados:**

Las CPO en el grupo ARISCAT con espirometría se presentaron en 11% de los pacientes, en comparación con el grupo ARISCAT sin espirometría (48%). Los pacientes ARISCAT sin espirometría de alto riesgo tuvieron una mortalidad más elevada (18%), en comparación con los pacientes que sí la realizaron (0.4%). Los resultados de la regresión logística indican que no realizar la espirometría preoperatoria incrementa la probabilidad de CPO y mortalidad.

**Conclusiones:**

Nuestros hallazgos sugieren que el uso combinado de espirometría preoperatoria y ARISCAT se asocia con menores CPO y mortalidad. Las guías clínicas futuras deberán recomendar el uso de la espirometría preoperatoria para pacientes con un nivel moderado o alto de ARISCAT en México.

## Introduction

Approximately 30% of the global burden of disease is attributable to conditions that require a surgical procedure.^1^ In countries such as Mexico, the SARS-CoV-2 pandemic had a major impact on the number of surgical procedures that were canceled due to competing roles and responsibilities among the clinical staff, resulting in a 30% decrease in surgical procedures from 2019 to 2020.^2^, ^3^ Of the surgical procedures performed between 2019 and 2020, 40% resulted in postoperative complications.^4^, ^5^, ^6^, ^7^, ^8^ The preoperative evaluation of patients aims to minimize risk factors and reduce postoperative complications (POC),^9^ through the use of various techniques and diagnostic tests. POC can increase postoperative morbidity and mortality by 15–30% and prolong hospital stay.^7^ The Assess Respiratory Risk in Surgical Patients in Catalonia (ARISCAT) is a clinical tool used for preoperative evaluation, which considers the following seven factors: age, pulse oximetry, respiratory infection within 30 days of surgery, preoperative anemia, site of surgery, duration of surgery, and whether the surgery is elective or emergency.^10^ A specific level is assigned to each patient based on a scale of low-, moderate-, and high-risk. The ARISCAT scale has been validated in a multicenter study and other research studies, which found an association between the three risk levels and an POC incidence of 3%, 13% and 38%, respectively.^11^, ^12^, ^13^

Preoperative spirometry is the gold standard for diagnosing airway flow obstruction and is a critical clinical tool to qualify the response to treatment over time.^14^ Some studies have found that spirometry helps to predict POC,^15^, ^16^ while other authors report that adequate treatment for bronchial asthma and COPD reduce POC.^17^, ^18^ The occurrence of POC in patients with abnormal spirometry can increase up to 30%, compared to patients with normal spirometry.^19^, ^20^

Despite the extensive evidence regarding the validity of the ARISCAT risk scale^10^, ^11^ and the benefits of preoperative spirometry,^14^, ^15^, ^16^, ^17^, ^18^, ^19^, ^20^, ^21^, ^22^ current guidelines for preoperative care in Mexico recommend that preoperative spirometry should only be performed on patients with a pulmonary disease diagnosis.^23^ Although the combination of preoperative spirometry and the ARISCAT scale has been found to improve patient outcomes by reducing POC and mortality,^10^, ^11^, ^12^, ^13^ this is the first study to evaluate this strategy in Mexico. Our study aimed to determine if preoperative spirometry can prevent POC and improve survival at each ARISCAT risk level at one of the largest public hospitals in Mexico City, the *Hospital General de México.*

We identified 2059 patients who had an ARISCAT assessment during the period of 2013–2017 and compared the incidence of POC and mortality in the groups with and without spirometry. Our hypothesis was that the combined use of preoperative spirometry and the ARISCAT scale would be associated with a reduced number of POC and lower mortality.

### Aim

The aim of this study was to compare the incidence of postoperative complications (POC), mortality, and risk factors among adults who did or did not undergo preoperative spirometry, based on their Assess Respiratory Risk in Surgical Patients in Catalonia (ARISCAT) risk level.

## Material and methods

### Study design and population

The research protocol for this observational, retrospective, and comparative study followed the Declaration of Helsinki's ethical requirements and was approved by the Institutional Review Board of the *Hospital General de México* (HGM) (DIR/18/503F/3/030). Informed consent was waived because this study involved the secondary analysis of patient medical records. Additionally, this study followed the STROBE guidelines for reporting observational studies.^24^

**Fig. 1:**
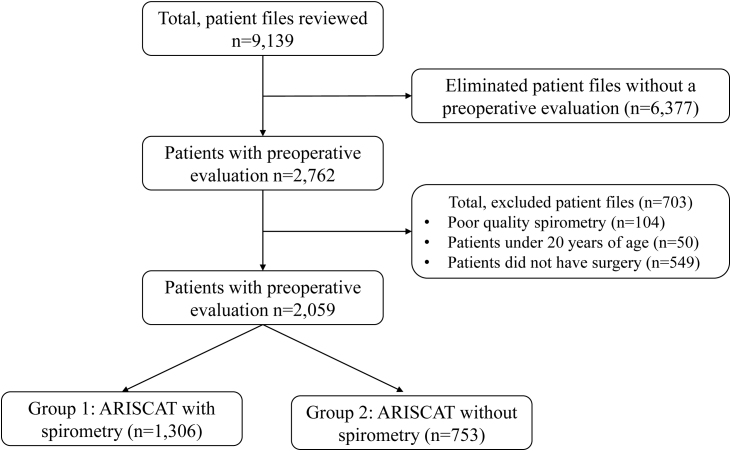
Eligibility criteria and final study sample based on level of ARISCAT risk and receipt of spirometry.

A total of 9139 clinical records were reviewed from the Department of Pulmonary Physiology and Hospitalization at the HGM in Mexico City, for the period from January 2013 to December 2017. The following inclusion criteria were used: adults aged 20 years or older, who underwent elective surgery, had an ARISCAT classification, and either received preoperative spirometry or did not, which resulted in 2762 eligible records. From these, 104 clinical records were excluded because the patients did not meet the required quality criteria for a spirometry evaluation, 50 patients were under 20 years of age, and 549 patients had their surgical procedure canceled (*n* = 703). The final sample of 2059 patients was further classified into two groups: Group 1, patients with an ARISCAT assessment who also had preoperative spirometry (*n* = 1306) and Group 2, patients with ARISCAT but no preoperative spirometry (*n* = 753). See Fig. 1.

### Types of elective surgery

Surgical procedures were classified based on the three anatomical regions, which corresponded to the ARISCAT risk classification criteria: (1) Thorax (e.g. cardiovascular surgery, esophageal cancer, lung cancer, breast cancer, surgeries for infectious chest sequelae, or non-oncological chest surgery); (2) Upper abdomen (e.g. non-oncological surgery of the upper abdomen, oncological surgery of the upper abdomen, or hiatal hernia repair); and (3) Peripheral (e.g. head and neck cancers, eye surgery, nervous system cancers, neck vascular surgeries, thyroid and thymus surgeries, thyroid cancer, facial reconstructive surgeries, lower abdominal oncological and non-oncological surgery, inguinal and scrotal hernia repair, orthopedic surgery, extremity oncological surgery, peripheral vascular surgery and reconstructive surgery). Surgical procedures in the abdominal or thoracic areas were classified as major surgery and all other surgeries were minor. Finally, pre-surgical diagnoses were classified as oncological and non-oncological surgeries.

### Preoperative clinical evaluation

Spirometry was performed with a Vmax22 equipment, Vyasis Healthcare, Yorba Linda, CA (USA), according to technical quality guidelines.^25^ Most patients received an initial spirometry evaluation, a bronchodilator challenge test, and had their ARISCAT risk level assessed within 30 days of their surgery date. In some cases, patients who were hospitalized before their surgery underwent these assessments on the actual day, or within a few days, of their surgical procedure. The spirometry diagnosis was defined as normal when the forced expiratory volume in one second (FEV_1_)/forced vital capacity (FVC) ratio was equal to or greater than the lower limit of normal according to the quality criteria of the American Thoracic Society. The spirometry diagnosis was classified as abnormal when it met the following criteria: (1) suggestive of restriction when the FEV_1_/FVC ratio was greater than the lower limit of normal but had decreased FVC, (2) when the FEV_1_/FVC ratio was less than the lower limit of normal and had decreased FEV_1_.^25^

Depending on the spirometry diagnosis, patients either received a preoperative medical treatment and proceeded with their surgical plan or were provided with specific recommendations for ventilatory practices during their surgery. Although the ARISCAT risk scale does not take into account other comorbidities, the status of patients with risk factors such as diabetes, hypertension, obesity, heart disease, smoking, neurological and rheumatological diseases was documented as part of their clinical evaluation in the pulmonary physiology department, at the time of their spirometry test and ARISCAT risk assessment.

### Postoperative care

The HGM has 1200 beds, of which 120 correspond to the seven Intensive Care Units (ICUs), and the hospital includes all clinical and surgical specialties. As a teaching hospital, the training of medical and nursing students, as well as residents and clinical fellows, is a routine part of the care of patients in the different surgical and clinical specialties. Most postoperative care occurs while patient recovers in general hospitalization beds. Only in specific cases such as chest surgery, cardiovascular or neurological surgery, or patients who had any complications in the intraoperative or immediate postoperative period receive their postoperative care in the ICUs. Postoperative care is generally provided by the doctors assigned per shift in all services and the doctors on duty at the different surgical specialties. If any complication is suspected, including the need for mechanical ventilation or hemodynamic support, a consultation is requested from the clinical teams working each shift and the specific indications for each case are followed, from adjusting medication, conducting additional clinical studies, or admission to the ICUs. In terms of nursing staff, one nurse is assigned per hospital area and in the ICUs, there is one nurse for every three patients. At the HGM, the careful follow-up care of patients is carried out throughout the postoperative hospitalization period.

### Study variables

The following variables were obtained from the patients’ medical records: sex, age, body mass index (BMI), history of smoking, exposure to biomass smoke, diabetes mellitus, abnormal spirometry results were recorded as lung disease, ARISCAT risk was classified as low (≤25 points), moderate (26–44), or high (≥45),^10^ surgical diagnosis, type of surgery, and specific type of POC observed: pulmonary,^11^ surgical, metabolic, cardiovascular, neurological, or vascular.

Pulmonary POC in our study were defined based on the criteria used in the PERISCOPE study, which considered the following complications: atelectasis, bronchospasm, pleural effusion, pneumonia, respiratory failure, pneumothorax and pulmonary embolism.^11^ Surgical POC included: abdominal pain, bleeding, fistula, hypovolemic shock, paralytic ileus, perforation, sepsis, and vascular injury. Cardiovascular POC included acute myocardial infarction and cardiogenic shock, metabolic POC included glycemic dysregulation, and hepatic or renal failure, neurological POC included acute vascular events, vascular POC included deep vein thrombosis.

All POC were evaluated and confirmed with the corresponding clinical studies that were included in the patient's medical records. We also confirmed that POC occurred during the subsequent hospitalization period and were due to the type of surgery performed; oncological or location of surgery; and whether surgery was major (thoracic or abdominal cavity surgery) or minor (skin, subcutaneous tissue and muscle wall), oncological, or non-oncological. Mortality was only considered as an outcome when it was directly linked to a specific POC that was associated with the patient's surgery.

### Statistical analysis

Patients who received spirometry (Group 1) were compared to patients who did not receive spirometry (Group 2) using the Chi-square tests for categorical variables and the Student's *t*-test for continuous variables. The Chi-square test was also used to compare the incidence of POC and mortality in the groups with and without spirometry, Fisher's exact test was used for subgroups with less than five patients and measures of central tendency and dispersion were also obtained. Multivariate logistic regression models were used to identify factors associated with increased POC and mortality. Variables included in the multivariate model were selected based on the statistical significance of the bivariate model (*P* < 0.05), multi-collinearity tests, and the clinical relevance of the predictor variables. The unadjusted and adjusted odd ratios (OR), along with their 95% confidence interval (95% CI), are reported. For all analyses, a two-tailed *P* < 0.05 was considered statistically significant. Statistical analyses were performed with SPSS IBM Statistics v.19 software.

## Results

### Population characteristics

The sociodemographic and clinical characteristics of Group 1: ARISCAT with spirometry (*n* = 1306), and Group 2: ARISCAT without spirometry (*n* = 753) are presented in Table 1. Of the total sample (*n* = 2059), 852 patients (41%) were male and 1207 (59%) were female. In Group 1: ARISCAT with spirometry, 930 patients (71%) had a normal result, and 376 (29%) patients received an abnormal result. The mean age of patients in Group 2 (50 years) was significantly lower than the mean age of patients in Group 1, 56.4 years with normal spirometry results and 59.6 years with abnormal spirometry.

**Table 1:** Characteristics of patients who had spirometry (Group 1) and those who did not (Group 2). *n* (%).

	Group 1 *n* = 1306				Group 2 *n* = 753	
	*A*	*B*	*A* vs B*	*C*	*D*	*C* vs D**
Variable	Normal spirometry *n* = 930	Abnormal spirometry *n* = 376	P*	Total *n* = 1306	Without spirometry *n* = 753	P**
*Sex*						
Male	330 (35)	146 (39)	0.000	476 (36)	376 (50)	0.000
Female	600 (65)	230 (61)		830 (64)	377 (50)	

*Age (median, SD)*	56.4 (±14.6)	59.6 (±14.7)	0.000	57.3 (±14.7)	50.0 (±15.9)	0.000

*Age*						
20–39 years	126 (13)	34 (9)	0.001	160 (12)	215 (29)	0.000
40–59 years	415 (45)	144 (38)		559 (43)	327 (43)	
≥60 years	389 (42)	198 (53)		587 (45)	211 (28)	

*BMI*						
<25 kg/m^2^ s	246 (26)	143 (38)	0.000	389 (30)	271 (36)	0.002
≥25 kg/m^2^ s	684 (74)	233 (62)		917 (70)	482 (64)	

*Smoking history*						
No	413 (44)	198 (53)	0.004	611 (47)	587 (78)	0.000
Yes	517 (56)	178 (47)		695 (53)	166 (22)	

*Biomass smoke exposure*						
No	491 (53)	183 (49)	0.177	674 (52)	720 (95)	0.000
Yes	439 (47)	193 (51)		632 (48)	33 (5)	

*Pulmonary disease*						
No pulmonary disease	882 (95)	–	0.000	882 (67)	–	
Asthma	48 (5)	50 (13)		98 (8)		
COPD	–	96 (25)		96 (8)		
Restrictive diseases	–	230 (62)		230 (17)		

*Diabetes mellitus*	90 (10)	31 (8)	0.41	121 (9)	102 (13)	0.002

*ARISCAT*						
Low	538 (57)	180 (48)	0.000	718 (55)	66 (9)	0.000
Moderate	278 (30)	122 (32)		400 (31)	92 (12)	
High	114 (13)	74 (20)		188 (14)	595 (79)	

*Anatomical site of surgery*						
Thorax	104 (11)	69 (18)	0.002	173 (13)	487 (65)	0.000
Upper abdomen	315 (34)	116 (31)		431 (33)	152 (20)	
Peripheral	511 (55)	191 (51)		702 (54)	114 (15)	

*Type of surgery*						
Non oncological	609 (65)	220 (59)	0.01	829 (63)	520 (69)	0.006
Oncological	321 (35)	156 (41)		477 (37)	233 (31)	
Minor surgery	233 (25)	97 (26)	0.77	330 (25)	60 (8)	0.000
Major surgery	697 (75)	279 (74)		976 (75)	693 (92)	

*Abbreviations*. ARISCAT: Assess Respiratory Risk in Surgical Patients in Catalonia risk score for postoperative complications. SD: standard deviation. COPD: Chronic Obstructive Pulmonary Disease. Peripheral surgery includes head, neck and limbs. Major surgery (thoracic or abdominal cavity surgery) or minor surgery (skin, subcutaneous tissue and muscle wall).

* *A* vs *B*: Chi-square test used to compare the normal and abnormal groups of Group 1 with spirometry.

** *C* vs *D*: Chi-square test used to compare the total of Group 1 with spirometry with Group 2 without spirometry.

Group 1 had a significantly higher proportion of females (64% vs. 50%), patients aged 60 years or older (45% vs. 28%), individuals who were overweight or obese (70% vs. 64%), smokers (53% vs. 22%), exposure to biomass smoke (48% vs. 5%), low or moderate ARISCAT risk (86% vs. 21%), peripheral surgeries (54% vs. 15%) and minor surgeries (25% vs. 8%) than Group 2. Group 2 had a significantly greater percentage of patients with diabetes (13% vs. 9%), a high ARISCAT risk (79% vs. 14%), thorax surgeries (65% vs. 13%), non-oncological surgeries (69% vs. 63%) and major surgeries (92% vs. 75) than Group 1 (Table 1).

### Comparison of POC and mortality in Group 1 and Group 2 patients

Table 2 compares the incidence of POC and mortality by ARISCAT risk in patients with spirometry (Group 1) and patients without spirometry (Group 2). A significantly greater amount of POC were observed among patients who did not undergo spirometry (48%) than those who did (11%). Nearly half of the patients who did not undergo a spirometry test were classified as having a high ARISCAT risk (*n* = 339). Pulmonary POC were the most frequent complications in all groups. Mortality was significantly lower among Group 1 patients (4%), compared with Group 2 patients (19%), with high-risk ARISCAT patients who did not have a spirometry evaluation (Group 2) having the highest mortality rate (18%).

**Table 2:** Incidence of postoperative complications (POC) and mortality by ARISCAT risk level, in patients with and without spirometry. *n* (%).

	Group 1 With spirometry *n* = 1306				Group 2 Without spirometry *n* = 753	
	*A*	*B*	*A* vs B*	*C*	*D*	*C* vs D**
	Normal spirometry *N* = 930	Abnormal spirometry *N* = 376	P*	Total *N* = 1306	Without spirometry *N* = 753	P**
**POC by ARISCAT**						
*None*	843 (91)	324 (86)	0.000	1167 (89)	391 (52)	0.000
*Low*	41 (4)	20 (5)		61 (5)	6 (1)	
*Moderate*	36 (4)	17 (5)		53 (4)	17 (2)	
*High*	10 (1)	15 (4)		25 (2)	339 (45)	

**Type of POC by ARISCAT**						
*None*	843 (91)	324 (86)	0.000	1167 (89)	391 (52)	0.000*
*Low*						
Pulmonary	19 (2)	14 (4)		33 (3)	2 (0.2)	0.013**
Other complications	22 (2)	6 (1)		28 (2)	4 (0.5)	
*Moderate*						
Pulmonary	18 (2)	13 (4)		31 (2)	15 (2)	
Other complications	18 (2)	4 (1)		22 (2)	2 (0.2)	
*High*						
Pulmonary	8 (0.8)	12 (3)		20 (1)	266 (35)	
Other complications	2 (0.2)	3 (1)		5 (0.3)	73 (10)	

**Type of POC by pulmonary disease group**						
*No pulmonary disease*						
Pulmonary	843 (90)	323 (86)	<0.000	1166 (89)		
Other complications	44 (4.7)	0		44 (3.4)		
*Asthma*	41 (4.1)	0		41 (3)		
Pulmonary						
Other complications	1 (0.1)	3 (1)		4 (0.3)		
*COPD*	1 (0.1)	2 (0.5)		3 (0.2)		
Pulmonary						
Other complications	0	13 (3)		13 (1)		
*Interstitial lung disease*	0	1 (0.5)		1 (0.1)		
Pulmonary	0	24 (6)		24 (2)		
Other complications	0	10 (3)		1 (0.1)		

**Mortality by ARISCAT**						
*Surviving*	905 (97)	356 (95)	0.000	1261 (96)	608 (81)	0.000
*Low*	10 (1)	12 (3)		22 (2)	2 (0.2)	
*Moderate*	11 (1)	6 (1)		17 (1.6)	10 (0.8)	
*High*	4 (1)	2 (1)		6 (0.4)	133 (18)	

*Abbreviations*. ARISCAT: Assess Respiratory Risk in Surgical Patients in Catalonia risk score for postoperative complications. COPD: Chronic Obstructive Pulmonary Disease.

* *A* vs *B*: Chi-square test and Fisher's exact test (*n* ≤ 5) were used to compare the normal vs. abnormal groups of Group 1 with spirometry.

** *C* vs *D*: Chi-square test and Fisher's exact test (*n* ≤ 5) were used to compare the total Group 1 with spirometry vs. Group 2 without spirometry.

Table 2 also presents a comparison of the group with normal spirometry results to the group with abnormal spirometry results. Patients with abnormal spirometry had a significantly greater incidence of POC (14% vs. 9%, respectively) and higher mortality (5% vs. 3%, respectively) than patients with a normal spirometry result. Pulmonary POC were significantly more frequent among patients with abnormal spirometry results. Patients with restrictive lung disease had the highest POC incidence.

Table 3 reports the results of the bivariate and multivariate correlates of POC and mortality. In the bivariate logistic regression analyses, the following factors were associated with a greater probability of POC: male sex, not receiving a spirometry evaluation, increasing ARISCAT risk, having major surgery, and surgery in the thorax or upper abdominal area. In the multivariate analyses, the risk factors for presenting POC include being male (OR = 1.79), not undergoing spirometry (OR = 3.81), an increasing ARISCAT risk level (OR = 1.03) and undergoing major surgery (OR = 2.39). Additionally, the bivariate results suggest that male sex, not having a spirometry procedure, increasing ARISCAT risk, having major surgery, oncology-related surgery, and surgery in the thorax or upper abdomen are related to an higher mortality risk. Lastly, the multivariate analyses indicate that not having spirometry (OR = 4.05), an increasing ARISCAT risk (OR = 1.02), and cancer-related surgery (OR = 1.69) are associated with a greater risk of death.

**Table 3:** Bivariate and multivariate correlates of postoperative complications (POC) and mortality. Odds ratios (95% confidence intervals).

	Postoperative complications		Mortality	
Variable	Model I	Model II	Model I	Model II
*Age (continuous)*	0.98*(0.98–0.99)	0.99 (0.98–1.00)	0.99 (0.98 –1.00)	1.00 (0.99–1.00)
*Sex (ref: female)*				
Male	1.91*(1.56–2.35)	1.79*(1.40–2.28)	1.50*(1.11–2.02)	0.81 (0.58 –1.12)

*Body mass index (ref: <25* *kg/m*^*2*^*)*				
≥25 kg/m^2^	0.69*(0.56–0.85)	0.83 (0.65–1.06)	0.61*(0.45–0.83)	0.75 (0.54–1.04)

*Smoking history (ref: no)*				
Yes	0.65*(0.52–0.80)	1.20 (0.91–1.57)	1.08 (0.80–1.47)	

*Biomass smoke exposure (ref: no)*				
Yes	0.29*(0.22–0.38)	0.86 (0.61–1.22)	0.30*(0.20–0.46)	0.76 (0.45 –1.28)

*Spirometry (ref: performed)*				
Not performed	7.77*(6.19–9.75)	3.81*(2.76–5.27)	6.68*(4.71–9.46)	4.05*(2.54–6.45)

*ARISCAT (continuous)*	1.05*(1.04–1.06)	1.03*(1.02–1.04)	1.04*(1.03–1.05)	1.02*(1.01–1.03)

*Surgery type (ref: minor)*				
Major surgery	3.92*(2.73–5.65)	2.39*(1.50–3.79)	2.92*(1.7–5.02)	1.50 (0.77–2.94)

*Surgery severity (ref: non-oncological)*				
Oncological	1.11 (0.90–1.37)		1.65*(1.22–2.23)	1.69*(1.21–2.36)

*Anatomical site (ref: peripheral)*				
Thorax/upper abdomen	3.79*(2.97–4.85)	0.95 (0.67–1.34)	2.95*(2.04–4.28)	1.00 (0.59–1.70)

Significant results are shown in bold text.

* *P* < 0.05 using logistic regression analysis. Model I is a bivariate analysis. Model II is a multivariate analysis, which includes the following variables: age, sex and the covariates that were significant in the bivariate analysis of each outcome variable. ARISCAT: Assess Respiratory Risk in Surgical Patients in Catalonia risk score for postoperative complications.

## Discussion

This study compared the risk factors as well as the incidence of POC and mortality among Mexican adults who did or did not undergo preoperative spirometry, based on their ARISCAT risk level. Our findings indicate that the combined use of spirometry and the ARISCAT risk assessment tool is an effective strategy to reduce POC and mortality in patients who are undergoing elective surgery in Mexico. Of particular significance is the fact that patients with a high-risk ARISCAT level who did not receive a spirometry assessment (Group 2) had the higher incidence of POC (45%) and mortality (18%), which is a clear indication that these patients are not being evaluated adequately prior to surgery. An explanation for this may be that because these patients did not undergo spirometry their preoperative risk was not determined, which resulted in a greater risk of POC, compared to patients who did undergo a spirometry assessment. Conversely, patients with a high-risk ARISCAT level who received a spirometry evaluation had a significantly lower incidence of POC (2%) and mortality (0.4%).

Patients with lung disease have an increased risk of POC, and several studies have reported that the incidence of POC among patients with lung disease can range from 19% to 40%.^17^, ^20^, ^21^, ^22^, ^26^, ^27^ In the present study, we found a lower rate of POC among the COPD patients who had a spirometry assessment (3%), compared to those without spirometry (35%). Since patients who did not undergo spirometry had no documented history of lung disease in their medical record, it is possible some of these patients may have had undiagnosed lung disease that was not being treated or accounted for prior to surgery, thus increasing their risk of POC.

A restrictive spirometry pattern is also associated with POC, which can range between 21.1% and 43.2%.^15^, ^28^ In our study, the incidence of pulmonary POC was highest among patients who did not receive a spirometry test and had a high ARISCAT risk level (35%). Of the POC we observed, pulmonary POC were more frequent among patients with restrictive diseases, compared to patients who had airflow obstruction. Our multivariate analyses indicate that being male, not undergoing spirometry, increasing ARISCAT risk, and having a major surgery were significantly associated with POC. Other studies found that being 65 years or older, receiving an abnormal spirometry result, undergoing upper abdominal surgery, being an active smoker, having lung disease, and a high or moderate ARISCAT risk level are significant risk factors for POC.^12^, ^13^, ^19^, ^29^, ^30^

The results of a recent study indicate that POC are associated with a 30-day mortality of 1.5% and a 90-day mortality of 2.8%.^31^ A literature review by Elmer et al. reports that after esophagectomy, mortality increased by 22% among patients with COPD, and spirometry was a good predictor of mortality risk.^32^ Gómez-Hernández et al. also found that nearly 23% of patients who had major complications and died after surgery were mainly due to respiratory failure.^33^ Schussler et al. reported a mortality of 19% in patients with COPD, depending on the extent of their lung resection.^34^ Our findings suggest that the use of spirometry, especially among high-risk patients, could help to reduce mortality by also lowering the probability of POC. Additionally, our result indicate that having an oncological surgery is a significant risk factor for increased mortality, and these findings are supported by other studies.^19^, ^26^, ^29^

This study has some limitations. The patient data used for this research was obtained from a single hospital, which limits the generalizability of our results. However, since all patient medical records were reviewed at the HGM (over 9000 records) to identify a sample of 2059 patients who had a preoperative evaluation and underwent surgery from 2013 to 2017, the internal validity of our findings is likely strong, especially regarding the patients undergoing surgery at the HGM. Additionally, we used the same criteria as the PERISCOPE study (Prospective Evaluation of a Risk Score for Postoperative Pulmonary Complications in Europe)^10^ to assess risk of postoperative pulmonary complications. This risk scale has strong internal and external validity because it is based on a large (*n* = 5099), prospective, and observational study that was conducted at 63 hospitals in 21 European cities.^11^ Also relevant, is the fact that spirometry is currently considered the gold standard to detect airflow obstruction. Nonetheless, future studies should evaluate the use of spirometry and the ARISCAT scale at other hospitals that are part of the Mexican Ministry of Health and other medical organizations in Mexico, to improve the generalizability and external validity of these results. Although some information about patient comorbidities was included in study (obesity, smoking history, pulmonary disease, and diabetes), we did not include other comorbidities such as cardiovascular disease, which is associated with an increased morbidity and mortality risk after surgery. Another limitation is the possibility that some of the high-risk patients at the HGM are not being referred for preoperative assessment due to the overscheduling of services at the Department of Pulmonary Physiology, and to prioritize surgical appointments. Our findings indicate that patients who received a spirometry evaluation had a lower ARISCAT risk than patients who did not undergo spirometry, who were more likely to have a high ARISCAT risk. Also, although current guidelines in Mexico recommend spirometry in patients with pulmonary disease, in our sample it was performed in 67% of patients with no pulmonary disease. This is because there is currently no triage system to prioritize patients who might be at higher risk of postoperative complications. While these limitations may result in a selection bias that may have affected our results, it also highlights the urgent need to develop better protocols for the preoperative evaluation of high-risk patients at the HGM. An aim of this study is to showcase the importance of systematizing the use of the ARISCAT risk scale to help facilitate the identification of high-risk patients who should undergo spirometry during their preoperative evaluation.

Despite these limitations, to the best of our knowledge, this comprehensive study was the first to examine the effectiveness of using preoperative spirometry and the ARISCAT risk scale to help reduce POC and mortality in Mexico. The ARISCAT scale is a low-cost clinical tool that is easy to perform and to interpret, since it accounts for factors that are strongly associated with POC, such as the site and duration of surgery. However, the ARISCAT assessment does not determine whether the patient has chronic pulmonary disease, because it only considers if the patient has had a respiratory infection within 30 days of surgery. Performing preoperative spirometry in patients who have a moderate or high ARISCAT risk is an effective way to assess the functional respiratory status of higher risk patients, since lung disease is associated with increased pulmonary POC, as well as cardiovascular and vascular POC.^35^ Two significant objectives are achieved by combining these two diagnostic tests, first, to improve the quality of preoperative care, and second, to ensure that the highest risk patients are undergoing preoperative spirometry to reduce the probability of POC and mortality. In addition, has also proven to be a cost-saving strategy.^36^, ^37^

Spirometry is a common preoperative test in high-income countries, but in low- or middle-income countries such as Mexico, it is still an accessible test only in second and third level hospital centers. As clinicians are faced with an increasing number of pulmonary complications among patients who had COVID-19, the combined use of spirometry and the ARISCAT risk scale is an important strategy to improve the quality of preoperative patient evaluations, reduce POC and mortality, and to optimize health care resources, especially in resource-limited countries such as Mexico. As evidenced by our results, we would suggest that the following clinical recommendations be implemented in Mexico: (1) Provide training and guidance to health personnel regarding the importance of conducting a preoperative assessment, which includes identifying the patient's ARISCAT risk level. (2) Perform preoperative spirometry in patients who have a moderate and high ARISCAT risk, or will be undergoing major surgery, or an oncology-related surgery. An ARISCAT assessment is simple, low-cost, and can be conducted quickly, which make it an ideal clinical tool to identify the patients who would benefit the most from preoperative spirometry. This first step would help to identify patients who might have respiratory problems, so that any underlying pulmonary disease could be managed prior to surgery, thus minimizing any postoperative risks and complications. These two actions will improve the quality of preoperative care and will lead to significant clinical benefit for surgical patients by identifying those who are at greatest risk of postoperative complications. In addition to improving patient outcomes, these recommendations will also help to optimize the use of resources, which are scarce in all health systems, but especially in low- and middle-income countries, like Mexico.

## Conclusions

In summary, our findings suggest that the combined use of preoperative spirometry and the ARISCAT scale can help to reduce POC and mortality. The findings of this study should be used to revise and update the current guidelines and health policies for preoperative procedures in Mexico. Preoperative spirometry should also be recommended for patients who have a medium or high ARISCAT risk level.

## Funding

This research did not receive any specific grant from funding agencies in the public, commercial, or not-for-profit sectors.

## Authors’ contributions

Yolanda Mares-Gutiérrez has contributed as principal investigator in the original idea, design of the study, administrative support, provision of study materials or patients, collection, assembly and data curation, analysis and interpretation, manuscript writing, review, and final approval.

Adrián Martínez-González has contributed as a collaborating author in the design of the study, administrative support, analysis and interpretation, manuscript writing, review and final approval.

Guillermo Salinas-Escudero has contributed as a collaborating author in the design of the study, analysis and interpretation, manuscript writing, review and final approval.

Manuel García-Minjares has contributed as a collaborating author in the data analysis and interpretation, manuscript writing, review, and final approval.

Stephanie Liu has contributed as a collaborating author in the review, and final approval.

Yvonne N. Flores has contributed as principal investigator in the original idea, design of the study, administrative support, provision of study materials or patients, collection, assembly and data curation, analysis and interpretation, manuscript writing, review, final approval, and corresponding author.

## Conflicts of interest

The authors declare no conflict of interest in submitting this manuscript for publication.
